# A Novel Deployment Scheme Based on Three-Dimensional Coverage Model for Wireless Sensor Networks

**DOI:** 10.1155/2014/846784

**Published:** 2014-06-17

**Authors:** Fu Xiao, Yang Yang, Ruchuan Wang, Lijuan Sun

**Affiliations:** ^1^Nanjing University of Posts and Telecommunications, Nanjing 210003, China; ^2^Jiangsu High Technology Research Key Laboratory for Wireless Sensor Networks, Nanjing 210003, China; ^3^Key Lab of Broadband Wireless Communication and Sensor Network Technology, Ministry of Education, Nanjing 210003, China

## Abstract

Coverage pattern and deployment strategy are directly related to the optimum allocation of limited resources for wireless sensor networks, such as energy of nodes, communication bandwidth, and computing power, and quality improvement is largely determined by these for wireless sensor networks. A three-dimensional coverage pattern and deployment scheme are proposed in this paper. Firstly, by analyzing the regular polyhedron models in three-dimensional scene, a coverage pattern based on cuboids is proposed, and then relationship between coverage and sensor nodes' radius is deduced; also the minimum number of sensor nodes to maintain network area's full coverage is calculated. At last, sensor nodes are deployed according to the coverage pattern after the monitor area is subdivided into finite 3D grid. Experimental results show that, compared with traditional random method, sensor nodes number is reduced effectively while coverage rate of monitor area is ensured using our coverage pattern and deterministic deployment scheme.

## 1. Introduction

With the development of wireless communication, sensor, and microelectromechanical system technology, wide application prospect is exhibited for wireless sensor networks, such as in military, environmental monitoring, health, medicine, smart home, and other areas, and it has shown a huge advantage especially in unmanned monitoring or harsh environment; thus wireless sensor networks act as an important basic perception network for internet of things (IOT). As one of the basic problems in infrastructure, widespread research interests have been attracted in coverage control and nodes' deployment. Research demonstrated that the deployment of sensor nodes reflects the cost and the performance of wireless sensor network by references [1, 2]. Reasonable deployment can obtain the optimum allocation of WSN resources; thus the perceived quality of the sensor network can be greatly enhanced. Given a sensor network, the coverage problem is a leading indicator to measure the effect of deployment, which reflects sensor node's monitoring level in designated surveillance area [3]. However, most existing works on sensor deployment mainly concentrate on ideal two-dimensional planes. Actually in real applications, sensors are deployed on three-dimensional environments.

In this paper, a three-dimensional coverage model and optimum deployment scheme are proposed for wireless sensor networks. The rest of this paper is organized as follows. In Section 2, related works are introduced. The three-dimensional coverage problem in wireless sensor network and optimum coverage pattern based on cuboids is proposed in Section 3. In Section 4, the relationship between coverage and sensor nodes' radius is deduced, and also the minimum number of sensor nodes to maintain network area's full coverage is calculated. Experimental results show the effectiveness of our method in Section 5, and conclusion is made in Section 6.

## 2. Related Works

The deployment scheme is one of the most important problems in wireless sensor networks, which act as the fundamental infrastructure for target monitor. According to the different methods of node deployment, it can be divided into random deployment and deterministic deployment. Deterministic deployment is characterized by calculating the precise location of all sensor nodes and then placing each sensor node to obtain optimum connectivity and coverage by using the minimum number in monitoring area whose size and characteristic are known. This deployment divides network into lattices and then sensor nodes are placed. Dhillon and Chakrabarty [4, 5] proposed the maximum average coverage algorithm and the maximum minimum coverage algorithm, which are based on current node deployment; sensor nodes are placed assignably in the grid through the use of optimized strategy to meet point's coverage requirements in each grid and ensure that the number of sensor nodes is minimized, while these node deployment schemes focus primarily on the ideal 2D plane, including the optimum deployment patterns based on rules polygon [6].

In fact, sensor nodes are in realistic three-dimensional physical world, whose perception model and corresponding perception of the scene are 3D structure. However, traditional simplified 2D perception model and its cover-control algorithm are difficult to directly be applied in realistic 3D physical environment [7, 8]. With the development of research work and the expanded demand for practical application, 3D sensor network attracts much attention, such as acoustic sensor network and atmospheric monitoring sensor network which are based on 3D scene [9]. Particularly in recent years, with the rise of underwater sensor network, researchers started to think about the sensor nodes' perception model and its corresponding deployment method which is closer to the realistic physics world. Ammari and Das [10] presented a distributed redeployment method for underwater sensor network by adjusting the position of nodes in the depth of the underwater to reduce the overlapping area, so as to achieve maximum coverage of the network. Alam and Haas [11] designed the Reuleaux tetrahedron model to characterize *k*-coverage of a 3D field and investigated the corresponding minimum sensor spatial density, considering the full coverage problem and the connection problem of 3D field, through calculating and comparing the common volume business of polyhydro. Reference [12] concludes that, when the ratio of node's communication radius to sensing radius is equal to or greater than a fixed value, the cover effect of truncated octahedron is better than that of cube, which is six-prism and rhombic dodecahedron. Amac Guvensan and Gokhan Yavuz [13] analyzed and summarized the directional coverage models of wireless multimedia sensor networks and thought that the sensing models of the sensor nodes for the three-dimensional space are the current hot spot. Liu and Ma [14] studied the coverage problem of wireless sensor networks for rolling terrains, and they derived the expected coverage ratios under the stochastic sensors deployment based on digital elevation model (DEM). Topcuoglu et al. [15, 16] studied positioning and utilizing sensors on a 3D terrain; however these works are not suited to 3D environments, such as atmospheric monitoring. And Kong et al. [17] studied the complex surface coverage problem in sensor networks.

The most related work is Zhang et al. [18], which studied the problem of constructing low-connectivity and full coverage for three-dimensional sensor networks. But they spend much work on ensuring *k*-connectivity (*k* ≤ 4) for the whole network. Our work is based on this, while we put more emphasis on deterministic deployment for sensor networks to ensure the three-dimensional space coverage. The Optimum coverage pattern and relationship between coverage and sensor nodes' radius are deduced in this paper. Experimental results show that, compared with traditional random method, nodes number is reduced effectively, while coverage rate of monitor area is ensured using our method.

## 3. Three-Dimensional Coverage Problem in Wireless Sensor Network

### 3.1. Problem Description

In wireless sensor network, we consider that all sensors are of the same type and have sphere-shaped sensing field with radius *r*_*s*_. 3D monitoring scene coverage can be abstracted as the ball-coverage problem, that is, covering a 3D space with several numbers of balls with the same radium, and each coverage area can be overlapped, and the requirement of completely coverage for monitoring area can be achieved finally. Considering the aim of prolonging network lifetime, optimization coverage problem corresponds to a specific problem that maximum covered a 3D space with least nodes.

### 3.2. Definitions

**Definition 1 (coverage pattern Ω).**

**Figure 1:**
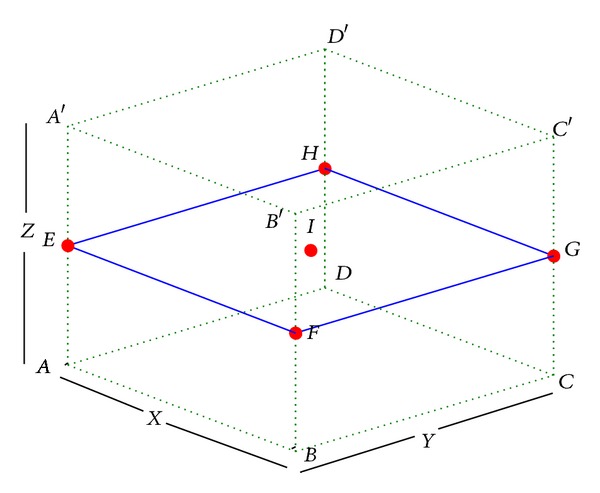
Coverage pattern Ω.

In three-dimensional space, a coverage pattern Ω is defined as in Figure 1, where *ABCDA*′*B*′*C*′*D*′ is a cuboid with length, width, and height as *x*, *y*, and *z* and its volume is denoted by *V*. Plane *EFGH* is located in the middle of cuboids *ABCDA*′*B*′*C*′*D*′. That is to say, it is parallel to the bottom *ABCD* at *z*/2 height and its center is point *I*.

Nodes deployment scheme is as follows: among five given points *E*, *F*, *G*, *H*, and *I*, a sensor node is placed at each position.

**Definition 2 (full coverage of coverage pattern Ω).**

Given a coverage pattern Ω and spheres with radius *r* centering at each point in five points *E*, *F*, *G*, *H*, and *I*, Ω is fully covered if every point in cuboid *ABCDA*′*B*′*C*′*D*′ can be covered at least by one node.

**Definition 3 (covering density).**

The ratio of the total volume of the five spheres with radius *r* to the volume of cuboid *ABCDA*′*B*′*C*′*D*′ is called covering density of Ω, which can be denoted by *σ*(*r*, *V*) as follows:

(1) $$\begin{array}{c} \sigma \left(r,V\right)=\frac{20\pi r^{3}}{\left(3V\right)}. \end{array}$$

**Definition 4 (optimum coverage pattern Ω′).**

Given sensing range *r*, a coverage pattern is called the optimum coverage pattern Ω′ if *σ*(*r*, *V*) is minimum among all coverage patterns which ensure the full coverage.

## 4. Establishment of Optimum Coverage Pattern

From Definition 4, to obtain the optimum coverage pattern for each case, the maximum volume of cuboid *ABCDA*′*B*′*C*′*D*′ which is denoted by *V*_max⁡_ should be obtained, as shown in (2), where *x*, *y*, *z* are the lengths of three orthogonal edges in the cuboid, as follows:

(2) $$\begin{array}{c} V_{\max}=xyz. \end{array}$$

**Figure 2:**
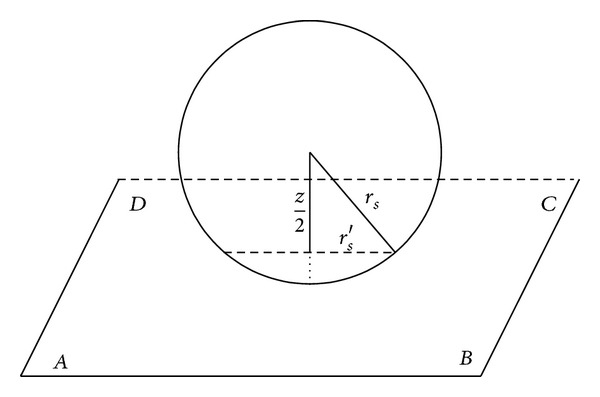
Coverage radius *r*_*s*_′ in plane *ABCD*.

*V*_max⁡_ can be obtained by solving a nonlinear optimization problem under constraints generated from full coverage. As shown in Figure 1, compared with other planes parallel to plane *EFGH*, plane *ABCD* and plane *A*′*B*′*C*′*D* are the hardest to cover since the intersections of sensing spheres on these planes are smaller than those on other parallelograms. If planes *ABCD* and *A*′*B*′*C*′*D* are covered, then any other parallelograms parallel to plane *EFGH* in this coverage pattern must be covered. According to the geometrical symmetry, we can map the full coverage of 3D cuboid to 2D rectangle *ABCD*. We suppose that the radius of sensor nodes is *r*_*s*_; then the sensing range of nodes is a circular area with the radius *r*_*s*_′ in plane *ABCD*. As Figure 2 illustrated, we can get

(3) $$\begin{array}{c} r_{s}^{\prime }=\sqrt{r_{s}^{2}-\frac{z^{2}}{4}}. \end{array}$$

The optimum full coverage of rectangle *ABCD* is obtained if the intersections of five sensor nodes' sensing sphere are smallest in rectangle *ABCD*. According to the relationship between length *x*, width *y*, and sensing radius, this problem can be divided into the following three cases.

**Case 1 .**

**Figure 3:**
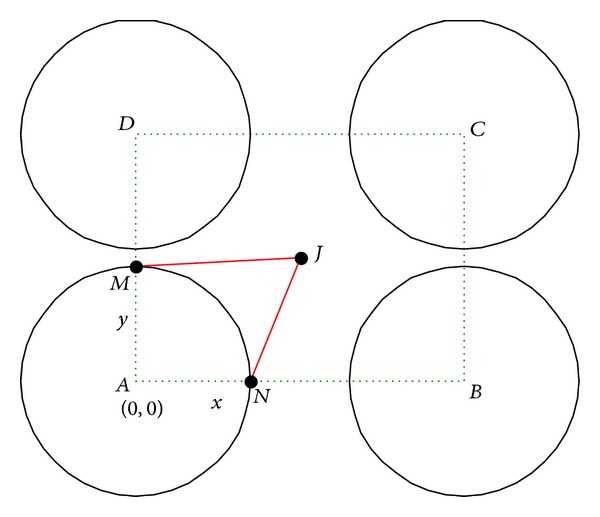
Case 1: relationship between *x* and *y*.

When *x* > 2*r*_*s*_′, *y* > 2*r*_*s*_′, as illustrated in Figure 3.

Let point *J* be the projection point of *I* at rectangle *ABCD*. Each sensing range of the sensor nodes at points *E*, *F*, *G*, *H*, and *I* is circular area with radius *r*_*s*_′ centering at each point in *A*, *B*, *C*, *D*, and *J*. Assuming that *A* is the origin point *O* at (0,0), *B* at (*x*, 0), *C* at (*x*, *y*), and *D* at (*o*, *y*), then we can get coordinates of *J* as (*x*/2, *y*/2), and |*AM*| = |*AN*| = *r*_*s*_′ is known. To ensure that the uncovered area in rectangle *ABCD* be covered by the circular area with radius *r*_*s*_′ centered at *J*, which is not covered by the other four circular areas with radius *r*_*s*_′ centering at *A*, *B*, *C*, *D*, the following conditions must be satisfied:

(4) $$\begin{array}{c} \left|JM\right|\leq r_{s}^{\prime },\\ \left|JN\right|\leq r_{s}^{\prime }. \end{array}$$

That is,

(5) $$\begin{array}{c} x^{2}+y^{2}-4x\sqrt{r_{s}^{2}-\frac{z^{2}}{4}}\leq 0,\\ x^{2}+y^{2}-4y\sqrt{r_{s}^{2}-\frac{z^{2}}{4}}\leq 0,\\ x>2\sqrt{r_{s}^{2}-\frac{z^{2}}{4}},\\ y>2\sqrt{r_{s}^{2}-\frac{z^{2}}{4}},\\ z<2\sqrt{r_{s}^{2}-\frac{z^{2}}{4}}. \end{array}$$

**Case 2 .**

**Figure 4:**
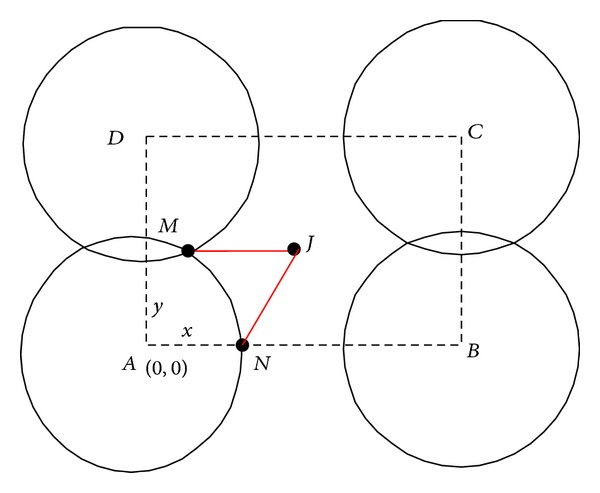
Case 2: relationship between *x* and *y*.

When *x* > 2*r*_*s*_′, *y* ≤ 2*r*_*s*_′, as seen in Figure 4.

Similarly, to ensure that rectangle *ABCD* be fully covered by five sensor nodes, these following conditions should be satisfied:

(6) $$\begin{array}{c} x^{2}+y^{2}-4x\sqrt{r_{s}^{2}-\frac{z^{2}}{4}}\leq 0,\\ x^{2}-y^{2}-4x\sqrt{r_{s}^{2}-\frac{y^{2}}{4}-\frac{z^{2}}{4}}\leq 0,\\ x>2\sqrt{r_{s}^{2}-\frac{z^{2}}{4}},\\ y\leq 2\sqrt{r_{s}^{2}-\frac{z^{2}}{4}},\\ z<2\sqrt{r_{s}^{2}-\frac{z^{2}}{4}}. \end{array}$$

**Case 3 .**

**Figure 5:**
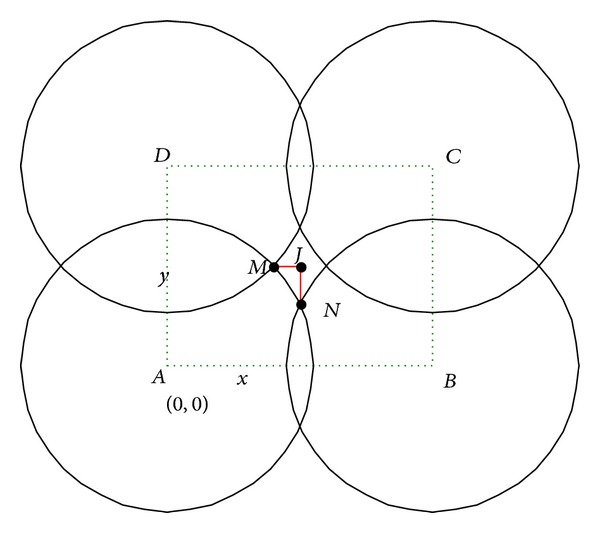
Case 3: relationship between *x* and *y*.

When *x* ≤ 2*r*_*s*_′, *y* ≤ 2*r*_*s*_′, as illustrated in Figure 5.

Similarly, these following conditions should be satisfied to ensure rectangle *ABCD* be fully covered by five sensor nodes:

(7) $$\begin{array}{c} x^{2}-y^{2}-4x\sqrt{r_{s}^{2}-\frac{y^{2}}{4}-\frac{z^{2}}{4}}\leq 0,\\ y^{2}-x^{2}-4y\sqrt{r_{s}^{2}-\frac{x^{2}}{4}-\frac{z^{2}}{4}}\leq 0,\\ x\leq 2\sqrt{r_{s}^{2}-\frac{z^{2}}{4}},\\ y\leq 2\sqrt{r_{s}^{2}-\frac{z^{2}}{4}},\\ z<2\sqrt{r_{s}^{2}-\frac{z^{2}}{4}}. \end{array}$$

Suppose the monitor environment is subdivided into cuboids with length *x*, width *y*, height *z*, and volume *V* and radius of sensor nodes is *r*_*s*_. By solving formula (2) under constraint as conditions (5), (6), and (7), we can obtain the optimum coverage pattern Ω′ as follows:

(8) $$\begin{array}{c} x=\frac{2\sqrt{6}r_{s}}{3},\\ y=\frac{2\sqrt{6}r_{s}}{3},\\ z=\frac{2\sqrt{3}r_{s}}{3},\\ V=\frac{16\sqrt{3}r_{s}^{3}}{9}. \end{array}$$

## 5. Simulations and Analysis

Suppose that the actual environment is a 3D rectangular with length *L*, width *W*, and height *H*, respectively. The cuboid owns space division character, and the real 3D physical environment can be subdivided into several meshes to achieve full coverage by adopting our optimal cuboid coverage pattern. To facilitate analysis, we ignore the region boundary in actual environment.

### 5.1. 3D Mesh Dissections on Actual Environment

**Figure 6:**
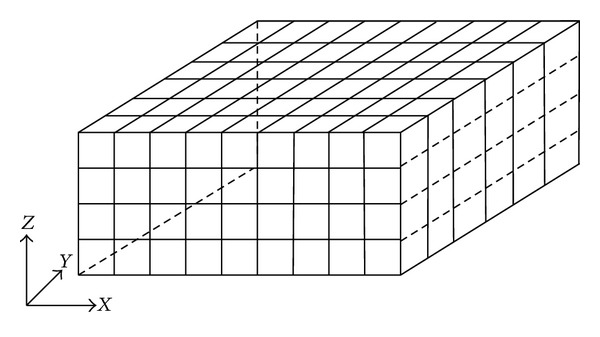
3D mesh dissections on the actual environment.

Based on structure size of the optimum coverage pattern, we subdivided the 3D rectangular into several small cuboids with length equal to $2\sqrt{6}r_{s}/3$, width equal to $2\sqrt{6}r_{s}/3$, and height equal to $2\sqrt{3}r_{s}/3$, as shown in Figure 6. We considered each little cuboid as an optimum coverage pattern and placed sensor nodes in Ω′. So the rectangular area is subdivided into cuboids as *ABCDA*′*B*′*C*′*D*′, and sensor nodes are deployed in the corresponding vertexes *A*, *B*, C, *D*, *A*′, *B*′, *C*′, *D*′ and point *I*. Number *N* of nodes to achieve full coverage of 3D scene can be calculated as follows:

(9) N=((⌈3L26rs⌉+1)×(⌈3W26rs⌉+1)+⌈3L26rs⌉) ×⌈H23rs/3⌉.

### 5.2. Performance Analysis

**Figure 7:**
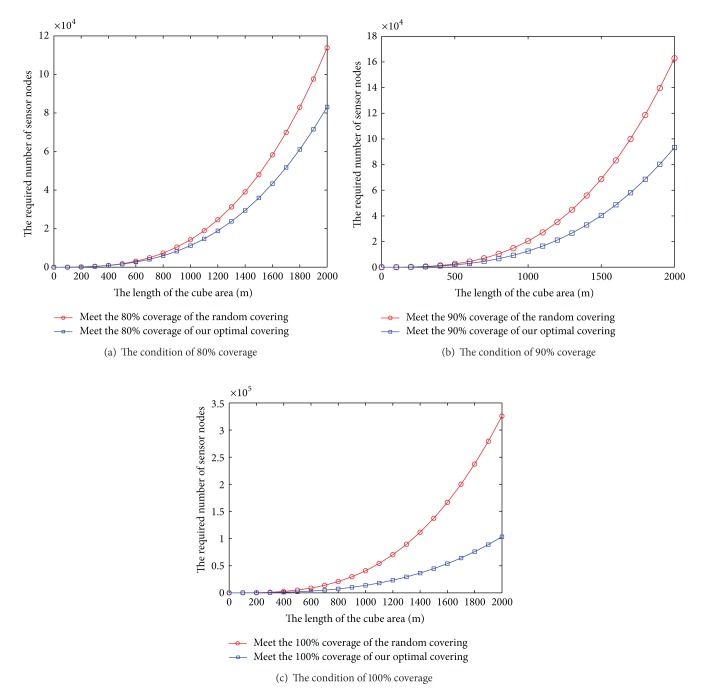
The condition of different coverage using our method and traditional random method.

We have conducted a series of simulation experiments in this given 3D monitor region to compare the differences between random deployment and deterministic deployment regarding required number of sensor nodes. For instance, deploying the sensor nodes in a cube with edge length 1000 m, when *r*_*s*_ = 30 m, we need around 14645 nodes to achieve full coverage of scene according to formula (5). We compare with random covering algorithm in the same area under the conditions of meeting the same coverage. The comparison results are shown in Figure 6 (meet 80% coverage in Figure 7(a), meet 90% coverage in Figure 7(b), and meet 100% coverage in Figure 7(c)). We can see that the deterministic deployment pattern needs smaller number of nodes than the widely used random deployment pattern under the conditions of meeting same coverage.

To validate the coverage influence of initial number of nodes on actual environment between random deployment and our deterministic deployment, we deploy the sensor nodes with *r*_*s*_ = 30 m in a cube with edge length of 1000 m and change the number of initial nodes. The results are shown in Table 1.

Table 1 illustrates that our deterministic deployment can make more coverage of actual area than random deployment under the condition of the same initial nodes. As illustrated by Zhang et al. [18], the constructing low-connectivity and full coverage three-dimensional sensor networks had been studied. Around 17200 nodes are needed to achieve 2-connectivity in a cube with edge length of 1000 m and sensor coverage radius equal to 30 m, which are the same as ours. While, in our scheme, we mainly focused on coverage and little attention had been paid to connective in sensor networks, no more than 15000 nodes are needed in our simulation results. Around 2200 nodes are reduced while coverage rate of monitor area is ensured using our method.

## 6. Conclusion and Future Work 

This paper mainly proposes a coverage pattern which is based on cuboid structure through analyzing the coverage characteristics of 3D scene. By deducing the required number of sensor nodes for full coverage in 3D monitor space theoretically, we get the deployment positions of sensor nodes based on our coverage pattern after subdividing the finite 3D grid triangulation of network area. A series of simulation experiments proves the availability of this deterministic deployment scheme and significant number of sensor nodes can be saved using this proposed scheme.

The results of this paper are carried out on the basis of 0-1 sphere sensing model, and future research can be carried out following these directions: (1) extending this 0-1 sphere sensing model to probabilistic sphere sensing coverage model; (2) mobile sensor and *k*-coverage deployment scheme. Moreover, connectivity problem is still an open interesting problem in three-dimensional deployment domains.

## Figures and Tables

**Table 1:** The influence of initial nodes number on the scene coverage rate.

Nodes number	Random strategy	Proposal strategy
10000	67.71%	68.60%
11000	71.16%	75.46%
12000	74.25%	82.32%
13000	77.00%	89.17%
14000	79.46%	96.03%
15000	81.65%	100%
16000	83.61%	100%
17000	85.37%	100%
